# Supplementary material for: The Role of the PAA1 Gene on Melatonin Biosynthesis in Saccharomyces cerevisiae: A Search of New Arylalkylamine N-Acetyltransferases
Source: Microorganisms. 2023 Apr 25;11(5):1115. doi: 10.3390/microorganisms11051115 (PMC10220653; doi:10.3390/microorganisms11051115)
Supplement: Supplementary file 1 [file microorganisms-11-01115-s001.zip › microorganisms-2354535-supplementary.docx]

Figure S1. Biosynthetic pathway for the synthesis of coenzyme A in yeast. Adapted from Liu et al. 2005 [18].


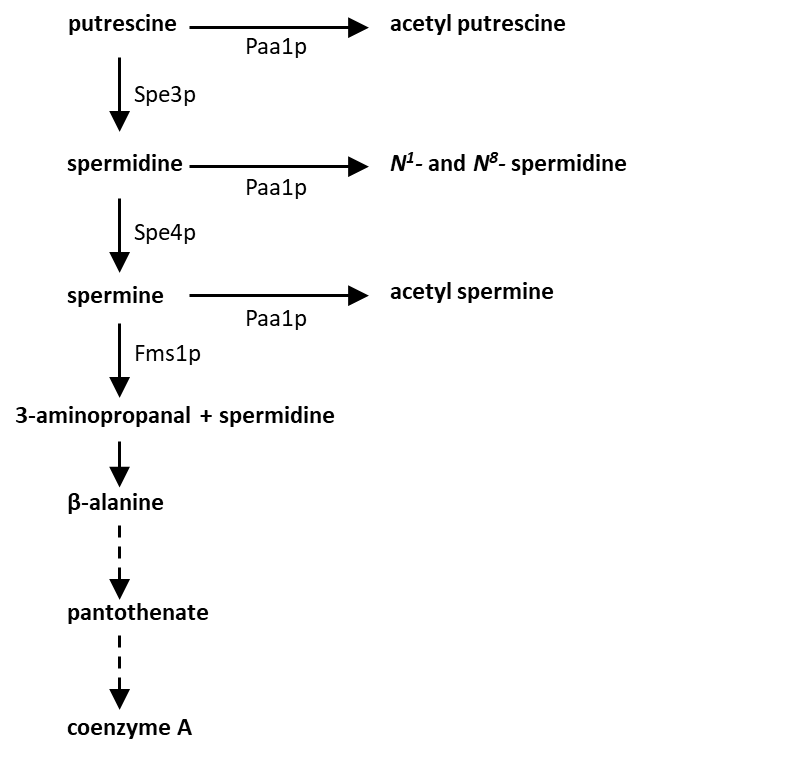


Table S1. Yeast strains used in this study.

| **Strain** | **Genotype** | **Source** |
| --- | --- | --- |
| BY4743 | *MATa*/*α his3Δ1*/*his3Δ1 leu2Δ0*/*leu2Δ0 LYS2*/*lys2Δ0 met15Δ0*/*MET15 ura3Δ0*/*ura3Δ0* | EUROSCARF |
| PAA1 | BY4743 with plasmid p426GPD *PAA1* (*URA3*) | This study |
| AANAT | BY4743 with plasmid p426GPD *AANAT* (*URA3*) | This study |
| fms1 | BY4743 *fms1Δ*::*KanMX* | EUROSCARF |
| paa1 | BY4743 *paa1Δ*::*KanMX* | EUROSCARF |
